# Bedside analysis of the sublingual microvascular glycocalyx in the emergency room and intensive care unit – the GlycoNurse study

**DOI:** 10.1186/s13049-018-0483-4

**Published:** 2018-02-14

**Authors:** Alexandros Rovas, Alexander-Henrik Lukasz, Hans Vink, Marc Urban, Jan Sackarnd, Hermann Pavenstädt, Philipp Kümpers

**Affiliations:** 1 Department of Medicine D, Division of General Internal Medicine, Nephrology, and Rheumatology, University Hospital Münster, Albert-Schweitzer-Campus 1, 48149 Münster, Germany; 2 Centre for Clinical Trials, University Hospital Münster, Münster, Germany; 3 Department of Physiology, Cardiovascular Research Institute Maastricht, Maastricht University, Maastricht, The Netherlands; 4 Department of Cardiology and Angiology, University Hospital Münster, Albert-Schweitzer-Campus 1, 48149 Münster, Germany

**Keywords:** Endothelial glycocalyx, Perfused boundary region, Intravital microscopy, Sidestream darkfield microscopy, Emergency room, Intensive care unit

## Abstract

**Background:**

Deterioration of the endothelial glycocalyx (eGC), a protective carbohydrate-rich layer lining the luminal surface of the endothelium, plays a key role in vascular barrier dysfunction and eventually organ-failure in systemic inflammatory response syndrome and sepsis. Early detection of glycocalyx damage could thus become an important goal in critical care. This study was designed to determine the feasibility and reproducibility of quantitative, real-time glycocalyx measurements performed at bedside in the emergency room (ER) and intensive care unit (ICU).

**Methods:**

The observational study included 70 patients admitted to the ER or ICU of a university hospital. A physician and the nurse in charge of the patient performed sublingual microcirculatory measurements using sidestream dark field (SDF) imaging. A novel data acquisition and analysis software (GlycoCheck™) was used to analyze the perfused boundary region (PBR), an inverse parameter of endothelial glycocalyx dimensions in vessels with diameters of between 5 and 25 μm.

**Results:**

The method showed a good intra-observer reproducibility. Specifically, intraclass correlation coefficient analysis showed an excellent reproducibility between the physician’s measurements (0.77 [CI 95%: 0.52–0.89]). The bias between the two PBRs was − 0.077 ± 0.24 μm. Moreover, there were no significant differences in the PBR values obtained by the nurses when compared to those reported by the physician (regarded as the “gold standard” measurement). Intraclass correlation coefficient analysis showed excellent reproducibility between the nurses’ and physician’s PBRs (0.75 [95% CI: 0.52–0.87]). The mean difference between the two PBRs (i.e., the bias) was 0.007 ± 0.25 μm. The nurses’ PBR assessment had a 90% sensitivity (95% CI: 60–99%) and 90% specificity (95% CI: 80–93%) to identify a severely impaired glycocalyx.

**Conclusion:**

Glycocalyx dimensions can be measured at patients’ bedside precisely by non-invasive assessment of the PBR. This assessment could become part of standard monitoring and contribute to clinical decision-making and resuscitation protocols in clinical trials and daily practice.

**Electronic supplementary material:**

The online version of this article (10.1186/s13049-018-0483-4) contains supplementary material, which is available to authorized users.

## Background

The endothelial glycocalyx (eGC) is a delicate gel-like layer coating the luminal surface of the vascular endothelium [1, 2]. It is up to 3 μm thick, largely consists of highly sulfated glycosaminoglycans and proteoglycans, and it plays a pivotal role in the maintenance of microcirculatory homeostasis [3, 4]. Specifically, the eGC acts as a negatively charged “firewall” to reduce leukocyte-endothelial-interactions [5]. Its carbohydrate-rich matrix provides resistance to water permeability (hydraulic conductivity) and contributes to the proportion of albumin molecules “reflected” back into plasma by the vessel wall (reflection coefficient) [6, 7]. Beyond that, the glycocalyx contributes to the regulation of the redox state and is crucially involved in the mediation of shear-induced nitric oxide release as well as physiologic anticoagulation [4, 8, 9].

The critical importance of the eGC has been highlighted in different vascular pathologies and particularly in the systemic inflammatory response syndrome (SIRS) and sepsis, where glycocalyx degradation plays a causative role in vascular barrier breakdown [10–12]. Recently, Schmidt et al. elegantly showed that inhibition of enzymatic glycocalyx digestion completely abolished acute lung injury and improved survival in a murine sepsis model [8].

Observational studies in critically ill patients have shown that the amount of shed eGC constituents – measured by enzyme-linked immunosorbent assay (ELISA) in blood samples – correlates with disease activity and predicts patient outcomes [13–17].

Recently, a novel automated acquisition and analysis software (GlycoCheck™) able to analyze the perfused boundary region (PBR), an inverse parameter of endothelial glycocalyx dimensions in sublingual microvessels, has become available [18]. Pilot studies conducted in the intensive care unit (ICU) revealed that the PBR is indeed markedly increased in critically ill patients compared to healthy controls [3, 19–22]. Whether context-specific PBR values, when measured early on in the emergency room (ER) can identify patients at high risk for organ failure and death, has not been determined. Thus, the present study was designed to assess the feasibility and reproducibility of PBR measurements under routine conditions.

## Methods

### Study population

This prospective, observational study was conducted in the University Hospital Münster. Patients were recruited from November 2016 to January 2017 in the interdisciplinary ER and in the 24-bed internal ICU. The study was performed in accordance with the Declaration of Helsinki and approved by the competent ethics committee (2016–073-f-S).

Seventy adult patients were enrolled upon presentation to the ER or admission to the ICU in a non-consecutive fashion after obtaining written informed consent from them or their legal representatives. Exclusion criteria were age < 18 years, pregnancy, and oral mucosal inflammation, which could locally compromise the sublingual glycocalyx. Demographic variables, routine chemistry tests, and physiological parameters, including the Sequential Organ Failure Assessment (SOFA) score [23], and a contemporary version of the Charlson Comorbidity Index (CCI) [24], were obtained for each subject immediately before the sublingual measurements (Table 1).

**Table 1:** Baseline characteristics

Variable	Total	ICU	ER	*P value*
Number of patients (n; %)	70 (100)	25 (35.7)	45 (64.3)	
Female sex (n; %)	37 (53)	15 (60)	22 (49)	0.46
Age (years, median (IQR))	61 (47.5–73.25)	63 (58–75.5)	58 (33.5–73)	0.07
BMI (kg/m^2^, median (IQR))	25.16 (22.29–28.48)	26.75 (24.89–32.16)	24.15 (21.98–26.28)	0.005
Diabetes Mellitus (n; %)	13 (19%)	6 (24%)	7 (16%)	0.52
CCI score (median (IQR))	2 (0–4)	2 (0.5–4)	1 (0–4)	0.51
SOFA score (median (IQR))	1 (0–2)	3 (1–6.5)	0 (0–1)	< 0.0001
Causes of admission/presentation				
Infection/Sepsis (n; %)	28 (40)	11 (44)	17 (37.8)	
ACS/Congestive heart failure (n; %)	11 (16)	8 (32)	3 (7)	
OHCA (n; %)	3 (4)	3 (12)	0 (0)	
Abdominal (n; %)	5 (7.1)	0 (0)	5 (11.1)	
Syncope/Arrhythmia (n; %)	7 (10)	0 (0)	7 (16)	
Other (n; %)	16 (23)	3 (12)	13 (29)	
Hemodynamic data (median (IQR))				
PBR (μm)	2.41 (2.26–2.61)	2.58 (2.29–2.72)	2.32 (2.24–2.53)	0.033
MAP (mmHg)	89.67 (75–101)	74.5 (70.67–85.5)	95 (86.33–106.7)	< 0.0001
Heart Rate (pulse/min)	79 (69–94)	85 (74.25–103.8)	79 (66–93)	0.12
Respiratory Rate (breaths/min)	19 (17.25–22.75)	21 (19–23.75)	18 (15–22)	0.004
Temperature (°C)	36.8 (36.5–37.18)	36.9 (36.53–37.28)	36.7 (36.5–37)	0.33
Laboratory data (median (IQR))				
WBC count (/μl)	9215 (7055–13,100)	12,730 (8755–15,510)	8060 (6205–10,720)	0.002
CRP (mg/dl)	2.25 (0.5–12.28)	9.8 (4–13.7)	0.9 (0.5–4.05)	< 0.0001
Lactate (mmol/l)	1.1 (0.9–1.7)	1.1 (0.9–1.55)	1.3 (1–1.7)	0.43

ACS = acute coronary syndrome, BMI = body mass index, CCI score = Charlson comorbidity index, CRP = C-reactive protein, IQR = interquartile range, MAP = mean arterial pressure, OHCA = out of hospital cardiac arrest, SOFA score = sequential organ failure assessment score, WBC = white blood cell

### Study design & measurements

In the first part of the study, a physician (A.R.) experienced in the use of the GlycoCheck™ System (Microvascular Health Solutions Inc., Salt Lake City, UT, USA) obtained two consecutive sets of sublingual measurements in 30 patients (20 in the ER and 10 in the ICU) to determine the intra-observer reproducibility. Each set consisted of two complete measurements (see below) which were averaged to account for spatial heterogeneity of the sublingual microvasculature.

In the second part of the study, the nurse *and* the physician each obtained a set of measurements in random order in 40 subjects (*n* = 25 in the ER and *n* = 15 in the ICU) to determine the inter-observer reproducibility. The nurses were blinded to the results obtained by the physician and vice versa. All measurements performed by the nurses were observed - but not assisted or corrected in any way - by the physician who timed the duration and judged real-time the overall quality (movement and pressure artifacts) of the videos on a 1 to 4 scale (1 = bad, 2 = moderate, 3 = good, 4 = excellent). All patients participating in the study were asked to assess the overall discomfort caused by the two sets of measurements using a 0 to 10 scale (0 = no discomfort, 10 = extreme discomfort). The two study groups were not different regarding CCI (*p* = 0.35) and SOFA score (*p* = 0.54), respectively (data not shown).

### Nurse training

Eight randomly invited nurses (division of acute and critical care) were trained to use the GlycoCheck™ System before the beginning of the study. In brief, all nurses underwent a theoretical training according to the current guidelines for optimal image acquisition and analysis of microcirculation [25], followed by an intensive hands-on training. This training was performed by an ER physician (A.R.). The nurses were shown how to recognize and avoid pressure and movement artifacts, which is a main concern in sublingual microvascular imaging [25]. In brief, it was recommended to pull the microscope back slowly until contact was lost and then slowly advance the probe again until contact was regained. At the end of the training, nurses’ videos were of good quality and all nurses were authorized to take part in the study.

### Imaging of microcirculation

**Fig. 1:**
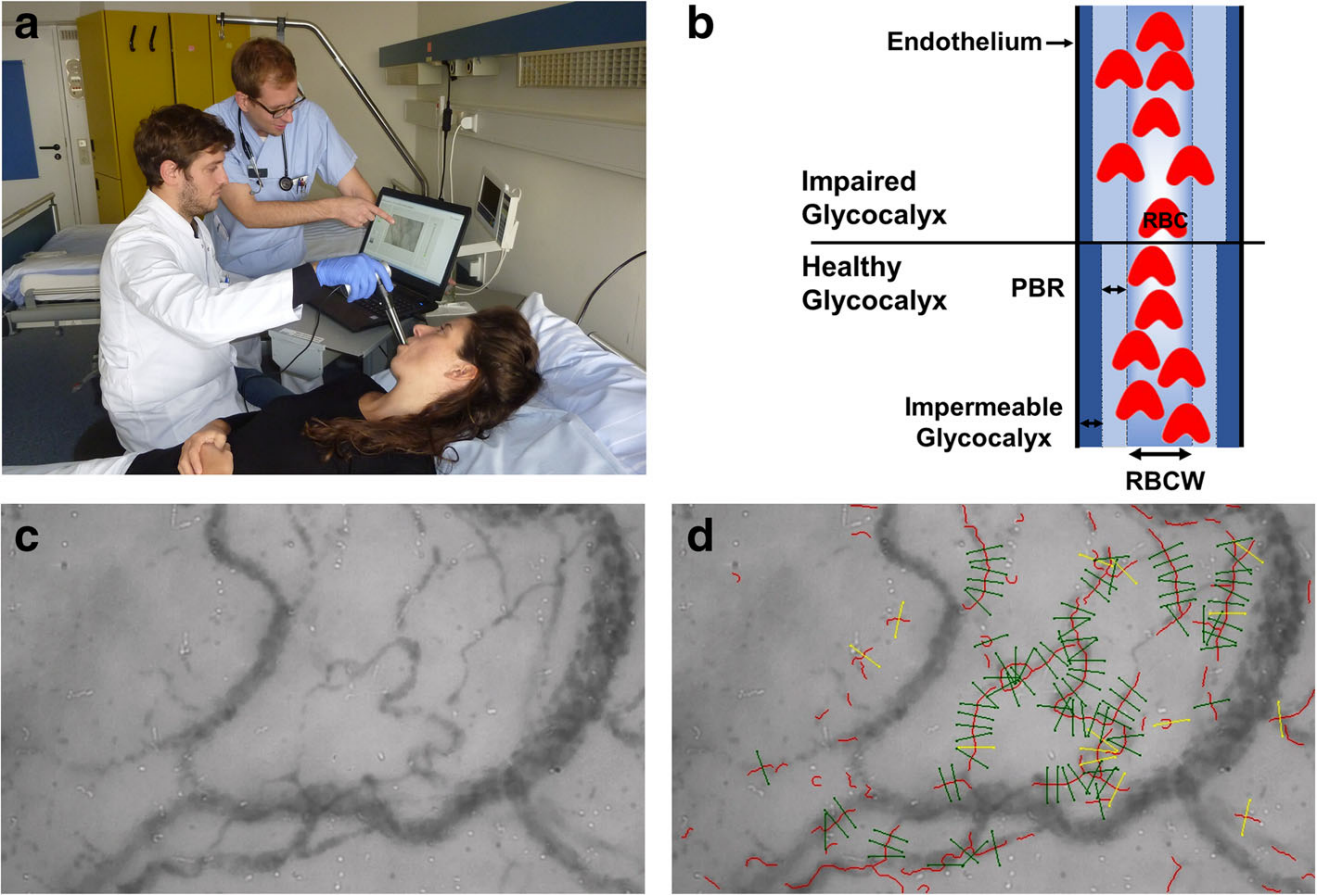
Image acquisition with the use of GlycoCheck™ System. Measurements were performed by using the GlycoCheck™ System, which consists of a sidestream-darkfield (SDF) camera coupled to a high-performance laptop computer. **a** A.R. (left) and P.K. (right) conducting a sublingual GlycoCheck™ measurement in a healthy volunteer. **b** Schematic illustration of cross section of a microvessel. GlycoCheck™ detects the dynamic lateral movement into the glycocalyx, which is expressed as the perfused boundary region (PBR, in μm). An impaired glycocalyx allows more RBCs to penetrate deeper towards the endothelial surface, which is reflected by an increase in PBR. **c** Representative image of the sublingual mucosa acquired with the SDF camera. **d** Quality check being automatically performed by the GlycoCheck™ software. Invalid vascular segments are marked yellow and are automatically discarded, while all valid vascular segments (green lines) are further analyzed. PBR: perfused boundary region, RBC: red blood cell, RBCW: red blood cell width

Real-time intravital microscopy was performed at the bedside with a sidestream dark field (SDF) camera (CapiScope HVCS, KK Technology, Honiton, UK) to visualize the sublingual microvasculature (Additional File 1). The SDF camera uses green light emitting stroboscopic diodes (540 nm) to detect the hemoglobin of passing red blood cells (RBCs). Using a 5× objective with a 0.2 numerical aperture, images were captured, providing a 325-fold magnification in 720 × 576 pixels at 23 frames per second as described in detail previously [3, 18, 20, 26–28]. Image acquisition and analysis was performed by GlycoCheck™ Software (Microvascular Health Solutions Inc., Salt Lake City, UT, USA) (Fig. 1a). It detects the dynamic lateral RBC movement into the glycocalyx, which is expressed as the PBR (in μm) [18]. A perturbed or degraded glycocalyx allows more RBCs to penetrate deeply toward the endothelial surface, with a consequent increase in the PBR (Fig. 1b).

Briefly, the software automatically starts recording the microvasculature when the criteria for high image quality (motion, intensity, focus) are fulfilled (Fig. 1c). It automatically identifies all available microvessels from 5 to 25 μm diameter and defines small vascular segments every 10 μm along the length of the detected vasculature. Subsequently, a sequence of 40 frames (i.e. 5-s video) is recorded containing, on average, 300 vascular segments (depicted as green lines in Fig. 1d). Then, the operator moves the camera to ~ 10 different position to record another 40 frames in each position. Once 3000 vascular segments have been captured, a measurement is completed. Each set (see “Study design & measurements”) consisted of two complete measurements which were averaged to account for spatial heterogeneity of the sublingual microvasculature.

After acquisition, the software performs a series of quality checks to validate that identified measurement sites indeed reflect straight segments of microvessels that contain a sufficient number of RBCs, automatically discards invalid vascular segments (yellow lines in Fig. 1d) and subjects all valid vascular segments (green lines in Fig. 1d) to further analysis. For each valid vascular segment, up to 840 radial intensity profiles are obtained, which are tested for the presence of RBCs. RBC filling percentage, signal quality, and RBC column widths are determined from these intensity profiles. This results in a RBC width distribution for each individual vascular segment from which the median RBC width as well as the outer edge of the RBC-perfused lumen (Dperf) is determined. The distance of the median RBC width (RBCW) value to the outer edge of the RBC-perfused lumen is measured and defined as the PBR (Dperf - RBCW)/2. Finally, the calculated PBR values, classified according to their corresponding RBC column width between 5 and 25 μm, are presented as a single median PBR score for each vessel diameter class and the corresponding 21 PBR values for diameter classes of 5 to 25 μm are averaged to provide a single PBR value for each participant. This method of calculating the PBR, which ensures that the average PBR value is equally weighted for each vessel diameter class, has been used and successfully validated previously [18, 27].

### Statistical analysis

Data are presented as absolute numbers, percentages, means with standard deviations, or medians with corresponding 25th and 75th percentiles (interquartile range; IQR) as appropriate. The non-parametric Wilcoxon matched-pairs signed rank test was used to compare PBR values between different measurements. The non-parametric Mann-Whitney U test and the Chi-square test were used to compare parameters between groups. To evaluate the *inter-* and *intra*-examiner reproducibility of PBR and RBC filling measurements, intraclass correlation coefficients (ICC) were calculated [29]. Specifically, the ICC (3, 2) with absolute agreement was calculated to analyze *intra-*examiner reproducibility, whereas *inter-*examiner reproducibility between nurses’ and the physician’s PBR measurements was analyzed using ICC (1, 2). ICC values were interpreted by the criteria suggested by Cicchetti et al. [30]: < 0.40 poor, 0.40 to 0.59 fair, 0.60 to 0.74 good, and > 0.74 excellent. The agreement between the two operators, nurse and physician, was visualized using the Bland–Altman method. The physician’s averaged PBR results served as the reference standard for this study. The sensitivity and specificity of the nurses’ PBR to detect a severely impaired glycocalyx (defined as a PBR ≥ 2.59 μm [3] measured by the physician) was calculated using contingency tables (www.statpages.info). To exclude potential heterogeneous performances between nurses, we calculated the delta of PBR measured by nurses and by the physician and used the Kruskal-Wallis test to compare the nurses. Correlations between variables were assessed using the Spearman rank correlation coefficient. Linear regression analysis was performed to investigate the association between PBR and RBC filling percentage. All tests were two-sided and significance was accepted at *p* < 0.05. Data analysis was performed using SPSS version 23 (IBM, Armonk, NY, USA). Figures were prepared using the GraphPad Prism version 7 (GraphPad Prism Software Inc., San Diego, CA, USA).

## Results

The clinical characteristics of the 70 subjects of the study are shown in Table 1. Our cohort had a balanced gender distribution with a median age of 61 years. The median [IQR] SOFA score was 1 [0–2], reflecting a rather low disease severity. The measurements were overall well-tolerated by the subjects with a median of score of 1 [0–2] on the standard analog discomfort scale of 0 to 10 points.

Regarding the microvascular parameters, there were no differences in either median PBR: 2.41 (2.13–2.63) vs. 2.53 (2.31–2.63) μm, *p* = 0.11; or RBC filling percentage: 61.75 (55.48–66.09) vs. 59.9 (55.74–63.73) %, *p* = 0.92 between the two subsequent sets of measurements performed by the physician (*n* = 30 patients). The ICC analysis, based on the Cicchetti Criteria, revealed an excellent intra-observer reproducibility for PBR of 0.77 (CI 95%: 0.52–0.89) μm and RBC filling percentage of 0.88 (CI 95%: 0.74–0.94), respectively. The Bland-Altman analysis showed a good agreement (Additional File 2: Figure S1). In fact, the two measurements taken by the physician were strongly correlated (r_s_ = 0.63, *p* < 0.0002).

**Fig. 2:**
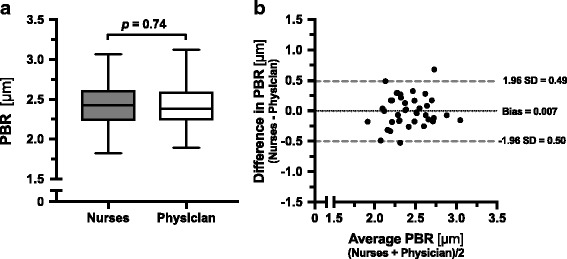
Inter-observer reproducibility of Perfused Boundary Region (PBR) measured by the nurses and physician. Eight trained nurses and a physician obtained paired sets of measurements (random order) in a total of 40 patients (*n* = 25 in the ER and *n* = 15 in the ICU) to determine the inter-observer reproducibility. **a** Boxplots showing PBR values (in μm) obtained by the nurses and the physician. The Wilcoxon signed-rank test was used to compare the paired PBR values. **b** Bland-Altman plot showing the limits of agreement (bias ±1.96 SD) between paired values for the nurses’ and physician’s perfused boundary region (PBR) measurements. (ER: Emergency Department, ICU: Intensive Care Unit, PBR: perfused boundary region, RBC: red blood cell

Each of the 8 nurses performed 4 to 7 sets of measurements in 40 additional patients to determine PBR and RBC filling values and evaluate the inter-observer reproducibility during routine care. Mean [± SD] duration of the bedside procedure (without calculations performed by the software) was 6 ± 3 min and the overall quality of the measurements performed by the nurses was evaluated as excellent with a median score of 4 (3–4) by the physician (A.R.). There were no differences between the nurses’ and the physician’s values for PBR: 2.43 (2.23–2.62) vs. 2.39 (2.24–2.59) μm, *p* = 0.74, and RBC filling percentage: 60.7 (53.99–64.45) vs. 60.18 (54.84–66.03) %, *p* = 0.45, respectively (Fig. 2a and Additional File 2: Figure S2). In fact, the subsequent measurements taken by the nurses and the physician were strongly correlated (r_s_ = 0.63, *p* < 0.0001). The ICC analysis showed excellent reproducibility between the nurses’ and physician’s PBR of 0.75 (95% CI: 0.52–0.87) and RBC filling percentage of 0.81 (95% CI: 0.64–0.90). Comparisons made using the Bland-Altman method showed a good agreement between the nurses’ and physician’s PBR assessment (Fig. 2b). The mean difference between the two PBRs (bias) was 0.007 ± 0.25 μm. There was no statistically significant heterogeneity between the performance of the different nurses (*p* = 0.12). The nurses’ PBR assessment had a 90% sensitivity (95% CI: 60–99%) and 90% specificity (95% CI: 80–93%) to identify a severely impaired glycocalyx (PBR ≥ 2.59, *n* = 10/40 patients – 3 patients in the ER group and 7 in the ICU group).

In a pooled analysis of the measurements from all 70 patients, the RBC filling percentage was associated with PBR (regression coefficient β: − 0.031 with 95% CI: − 0.037 to − 0.024), and it explained 59% of the variability in PBR (R^2^: 0.59, *p* < 0.0001) after adjustment for age, gender, BMI and disease severity (SOFA score) (Table 2 and Additional File 2: Figure S3). The median PBR was higher in ICU: 2.58 (2.29–-2.72) than in ER subjects: 2.32 (2.24–2.53), *p* = 0.033 (Additional File 2: Figure S4). Furthermore, PBR was moderately correlated with several markers of critical/acute illness including mean arterial pressure (MAP) (r_s_ = − 0.33 with 95% CI: -0.54 to − 0.09 and *p* < 0.01), C-reactive protein levels (r_s_ = 0.35 with 95% CI: 0.12 to 0.54, and *p* < 0.005), and SOFA score (r_s_ = 0.29 with 95% CI: 0.04 to 0.5 and *p* < 0.05). No correlations were found between PBR and age, gender or comorbidity.

**Table 2:** Regression Coefficient β

Independent Variable*	Regression Coefficient β	R Square	95% CI for coefficient β
RBC filling percentage	− 0.031	0.59	− 0.037 to − 0.024
+Age, sex, BMI, SOFA	−0.03	0.609	−0.037 to − 0.024

*Dependent Variable: Perfused boundary region (PBR)

*BMI*, body mass index, *RBC* filling percentage, red blood cell filling percentage, *SOFA score*, sequential organ failure assessment score

## Discussion

Our study is the first evaluating and reporting inter- and intra-observer reproducibility of the GlycoCheck™ method under routine clinical conditions. Moreover, it demonstrates that a real-time, bedside evaluation of the endothelial glycocalyx can be performed by trained nursing personnel in the ER and ICU.

Analysis of the microcirculation in critically ill patients has been recognized as a novel approach to improve risk stratification, prognostication, and individual therapy. For example, De Backer et al. [31] found that the proportion of perfused small sublingual vessels outperformed global hemodynamic variables to predict ICU mortality in 252 patients with severe sepsis. However, the approach and the methodology we used are different from previous studies, which analyzed quite different parameters (e.g. vessels density, proportion of perfusion, and various flow indices) using relatively short video sequences from few sublingual positions in a delayed fashion (i.e. off-line analysis). In contrast, the GlycoCheck™ software focuses on glycocalyx dimensions. It automatically captures twenty 5-s-videos from different positions – only after the predefined quality criteria (focus, intensity, motion) are being fulfilled. So far, real-time glycocalyx analysis was only performed in smaller studies of up to 50 individuals [3, 14, 19–22]. Although these studies extend experimental findings about the pathophysiological importance of the glycocalyx in critical illness, large-scale trials investigating the predictive performance of glycocalyx impairment have not been reported yet. This may be because PBR measurement remains an investigative procedure, performed by a limited number of experienced clinicians.

Our data indicate that PBR and RBC filling percentage can be reliably measured at the bedside. We believe that PBR values can be used to roughly differentiate a healthy from an impaired glycocalyx, despite spatial variability of the microvasculature. Indeed, the nurses’ measurements in our study had high specificity (90%) and sensitivity (90%) for detecting a severely impaired glycocalyx. This finding is in line with a recent report by Tanaka et al., who showed that trained ICU nurses can precisely determine vascular density and microvascular flow index of sublingual microcirculation [32]. Although both studies evaluated the feasibility and precision of bedside videomicroscopy performed by trained nursing personnel, each of them focused on different parameters being assessed with different software.

There are some limitations of this study. Firstly, it was from a single center, comprised of a heterogeneous set of patients with various diagnoses and rather low disease severity, and was not designed to evaluate PBR values with respect to specific disease outcomes. However, we believe that our results are representative as variability was relatively stable across the whole range of disease severity. Future studies should focus on specific disease entities (e.g. sepsis) in larger multicenter studies. Secondly, the sample size of this pilot study is limited and only 8 nurses participated in the study. To the best of our knowledge, the only comparable study by Tanaka et al. [32] had a sample size of 20 ICU patients. However, we cannot exclude that, if implemented in routine care and performed by the entire nursing team, variability might be higher than in the current study. Thirdly, although the supervising physician ensured that the quality of the videos made by the nurses was good, we cannot exclude bias due to pressure artifact, which is a concern in sublingual videomicroscopy. The good reproducibility between nurses’ and physician’s RBC filling percentages (as an indirect measure of pressure) argues against this hypothesis. Furthermore, we found a close inverse correlation between RBC filling percentage and the PBR in our study. This finding corroborates results from Lee et al. [18] who found that an impaired, permeable glycocalyx (high PBR) allows distribution of the RBCs in a bigger intravascular volume, resulting into a lower RBC filling percentage and a poor tissue perfusion. Fourthly, it is possible that the variability between the measurements is due to sampling error. However, we tried to counterbalance this by performing two complete measurements per set (about 20 different positions), which were averaged to account for spatial heterogeneity of the sublingual microvasculature. Moreover, the paired sets by the physician and the nurses were deliberately performed in random order.

Generally, sublingual PBR measurements were very well-tolerated by the patients in the current study. The procedure requires a low level of patient co-operation, allowing for the use of the GlycoCheck™ System in almost all individuals, even in intubated patients. Possible exceptions could be inflammation of the sublingual mucosa (e.g., mucositis in oncologic patients) as well as severe movement artefacts (e.g., in hyperactive delirium or intoxication), which could preclude high-quality recordings. In our experience, the PBR can be measured in about 95% of patients from the ER and ICU. Hence, we believe that monitoring of the endothelial glycocalyx can be successfully added to the clinical routine.

## Conclusion and outlook

We found that the GlycoCheck™ System is a reliable, feasible method to analyze glycocalyx properties when performed by trained nursing personnel. It is conceivable that *PBR screening* (e.g., at ER admission) might reveal a subgroup of septic patients without apparent end organ-failure (and not yet meeting the Sepsis-3 definition of sepsis) but with overt glycocalyx damage. Given the high chance of further deterioration, such patients might benefit from early, intensive monitoring. Therefore, a prospective, observational study to define PBR cut-offs for risk prediction is currently running in our hospital (*E*arly *D*etection of *G*lycocalyx Damage in *E*mergency Room Patients (EDGE Study, Clinicaltrial.gov Identifier: NCT03126032)).

## Additional files

Additional File 1: Standard Operating Procedure (SOP) (DOCX 16 kb)

Additional File 2: Figures (DOCX 238 kb)

## Abbreviations

BMI: Body mass index
CCI: Charlson comorbidity index
eGFR: Estimated glomerular filtration rate
ER: Emergency room
ICC: Interclass correlation coefficient
ICU: Intensive care unit
IQR: Interquartile range
LDH: Lactate dehydrogenase
MAP: Mean arterial pressure
PBR: Perfused boundary region
RBC: Red blood cell
RBCW: Red blood cell width
SDF: Sidestream dark field
SIRS: Systemic inflammatory response syndrome
SOP: Standard operating procedure
UKM: University Hospital Münster
WBC: White blood cell
ZKS Münster: Centre for Clinical Trials Münster
