# Supplementary material for: Bedside analysis of the sublingual microvascular glycocalyx in the emergency room and intensive care unit – the GlycoNurse study
Source: Scand J Trauma Resusc Emerg Med. 2018 Feb 14;26:16. doi: 10.1186/s13049-018-0483-4 (PMC5813422; doi:10.1186/s13049-018-0483-4)
Supplement: Supplementary file 1 — Standard Operating Procedure (SOP) (DOCX 16 kb) [file 13049_2018_483_MOESM1_ESM.docx]

**Additional File 1**

**Standard Operating Procedure (SOP)**

**Measurement of the sublingual endothelial glycocalyx**

**with the use of GlycoCheck^TM^ System**

1. Plug and turn the GlycoCheck^TM^ System on after connecting the SDF Camera on the USB port on the right side of the laptop.
2. Turn on the GlycoCheck software and calibrate the SDF camera before the first use.
3. Put lens cover tip on the camera.

1. Check the oral mucosa for signs of mucositis or oral injury. If signs of mucositis or oral injury are present, the procedure is being interrupted.

1. If needed, gently eliminate oral secretions with the use of wet swabs.

1. Place the SDF camera sublingually and start the measurement.

1. Look for representative sites of the oral mucosa and adjust focus if needed.

1. To avoid pressure artifacts, the microscope should be pulled back slowly until contact is lost and then slowly the probe should be advanced again until contact is regained.
2. When the criteria for high image quality are fulfilled (motion, intensity, focus), the video is being automatically acquired.

1. Slowly move the camera to another neighboring position of the sublingual mucosa and remain there for 5 seconds.
2. Repeat steps 6 to 9 until the measurement is complete (about 10 different positions/videos).
3. Start next measurement after calculation is complete (~2 minutes).
4. When done, shut down the computer, dispose the cover tip and disconnect the camera.
