# Supplementary material for: Bedside analysis of the sublingual microvascular glycocalyx in the emergency room and intensive care unit – the GlycoNurse study
Source: Scand J Trauma Resusc Emerg Med. 2018 Feb 14;26:16. doi: 10.1186/s13049-018-0483-4 (PMC5813422; doi:10.1186/s13049-018-0483-4)
Supplement: Supplementary file 2 — Figures (DOCX 238 kb) [file 13049_2018_483_MOESM2_ESM.docx]

**Additional File 2**

**Figures**

**Figure 1: Intra-observer reproducibility of PBR measurements.** Bland-Altman plot showing the limits of agreement (bias ± 1.96 SD) between paired values for the two physician’s PBR sets.

**Figure 2: Inter-observer reproducibility of RBC filling (%) measured by nurses and physician.** Eight trained nurses and one physician obtained paired sets of measurements (random order) in a total of 40 patients (n = 25 in the ER and n = 15 in the ICU) to determine the inter-observer reproducibility. Boxplots showing RBC Filling values (in %) obtained by the nurses and the physician. The Wilcoxon signed-rank test was used to compare the paired PBR values.

**
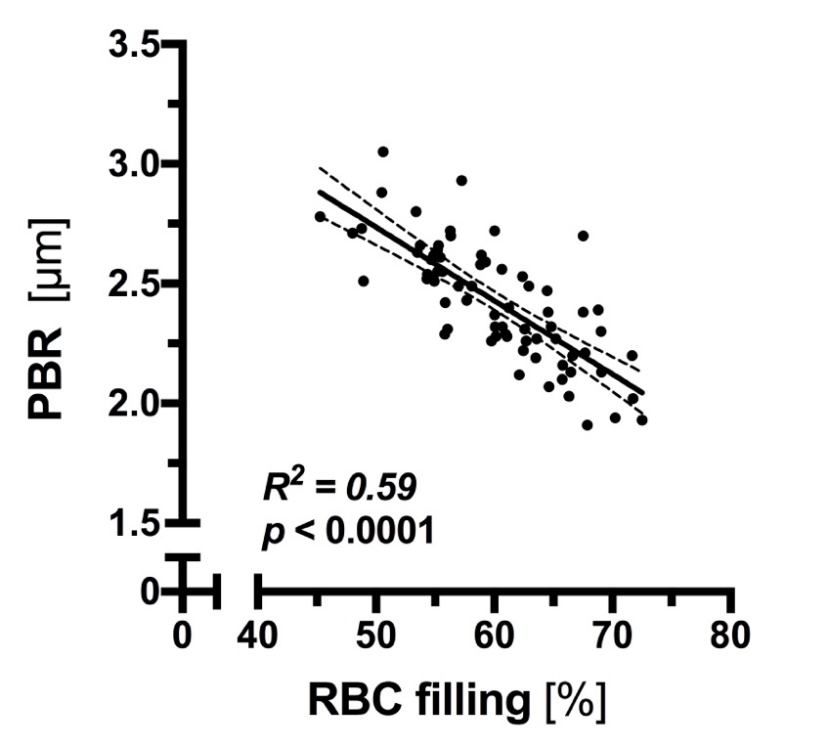
**

**Figure 3: Spearman correlation between PBR values and RBC values of the 70 patients participating in the study.** (R_s_ = - 0.77, *p* < 0.0001 // R^2^= 0.59, *p* < 0.0001).


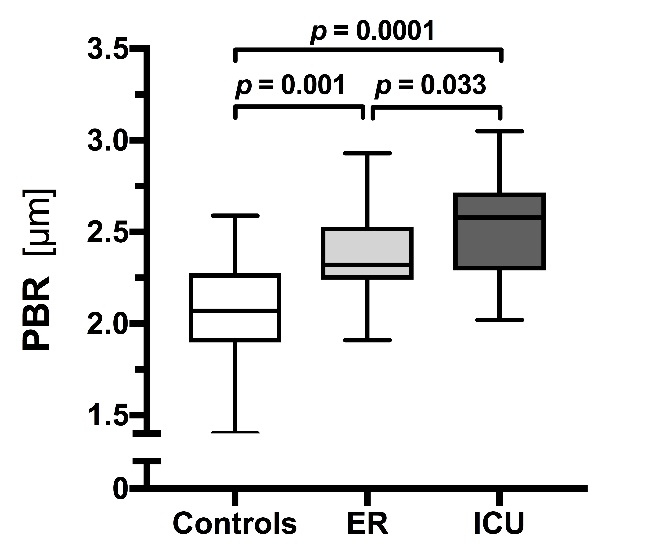


**Figure 4: Comparison of PBR values between healthy controls, ER and ICU patients.** Boxplots of PBR values (in μm) of healthy controls (n = 12), subjects in the ER (n = 45) and ICU (n = 25). Twelve unmatched healthy controls (5 females – median age 28 years old [25 to 37]) are shown as an additional control group (PBR 2.07 μm [1.9-2.28] µm). The Mann-Whitney U test was used to compare the PBR values between the groups.
